# Capacity-building interventions for health extension workers in Ethiopia: A scoping review

**DOI:** 10.1371/journal.pone.0317198

**Published:** 2025-01-13

**Authors:** Tigist Astale, Helina Mesele, Sarah-Louise Pasquino, Anteneh Zewdie, Eskinder Wolka, Aklilu Endalamaw, Yibeltal Assefa, Getnet Mitike

**Affiliations:** 1 International Institute for Primary Health Care–Ethiopia, Addis Ababa, Ethiopia; 2 Healthnet Internal Medicine Specialty Center, Addis Ababa, Ethiopia; 3 The University of Queensland, Brisbane, Australia; Management Sciences for Health (MSH), ETHIOPIA

## Abstract

**Introduction:**

Capacity-building interventions for health extension workers (HEWs) are key to providing quality health services to the community. Since Ethiopia’s Health Extension Program was established, several types of capacity-building interventions have been developed to build HEW competencies. However, no comprehensive study has mapped the types of capacity-building interventions being used or the competencies targeted.

**Objective:**

To (1) identify and characterize evidence on capacity-building interventions for Ethiopian HEWs, including the competencies measured; (2) clarify evidence gaps in this area; and (3) explore how successful the interventions have been to inform the design of health extension programs and further research.

**Methods:**

We used keywords (health extension workers, capacity building, competencies) and related terminologies to search PubMed, Scopus, and Embase for published studies on capacity-building interventions for Ethiopian HEWs, and Google Scholar for unpublished studies and reports. Our search was limited to studies and reports published in English from 2003 to present. We used the JBI scoping review methodology to conduct this scoping review in a stepwise approach and a categorization approach to synthesize the evidence.

**Results:**

Our search strategy identified 20 articles, all published except for one program report. The most common capacity-building intervention designed for HEWs was training, followed by supportive supervision, performance review and clinical mentoring meetings, and equipment supply; the most salient competency domains investigated were knowledge and skills. The interventions significantly improved immediate outcomes (knowledge, skills, attitude change among HEWs) and intermediate outcomes, such as increased service utilization and health-seeking behavior among community members. Only one study assessed whether capacity-building interventions improved inter- and intra-personal domains of capacity/competency.

**Conclusions:**

Capacity-building interventions for Ethiopian HEWs were found to be effective, but they mainly focused on improving technical competencies, such as knowledge and skills. Little attention has been paid to other competency domains, including motivation, leadership, and communication. Thus, future research could focus on a comprehensive set of capacity-building initiatives that addresses motivation, job satisfaction, communication, commitment, and resource allocation.

## Introduction

A robust health workforce is critical in order for health systems to function optimally and to achieve universal health coverage [1]. Community health workers (CHWs) are vital to extending services to underserved populations. Particularly in resource-constrained settings, CHWs are deployed where community members live to ensure the right to health [2]. In Ethiopia, CHWs are known as health extension workers (HEWs) and have been deployed since 2003 [3]. HEWs are the first point of contact with health services for the community and provide health promotion and preventive services, as well as selected curative services [4]. Deployment of HEWs as part of Ethiopia’s Health Extension Program (HEP) has enabled the country to significantly improve health service utilization, hygiene, and sanitation [5, 6].

However, the mere availability of HEWs may not be sufficient to achieve universal health coverage in Ethiopia. Studies have shown that Ethiopian HEWs’ competencies are suboptimal in regards to providing antenatal and delivery care [7] and newborn care [8], classifying common childhood illnesses [9, 10], and adhering to treatment guidelines [11], resulting in an overall gap in providing quality care [8]. A systematic review conducted in 2018 also identified a major challenge facing the HEP as HEWs’ lack of competencies in terms of knowledge, skills, and confidence [5].

Providing capacity-building interventions for the health workforce is considered a core strategy for promoting quality health service delivery, both at a national and global scale [1, 4]. Traditionally, it was widely believed that health workers’ poor performance resulted from lack of knowledge and skills [12]. This notion has been refuted by evidence suggesting health workers’ long-term performance is affected by various interlinked factors, such as knowledge, skills, motivation, confidence, personal goals, and guideline complexity [12]. Accordingly, interventions to improve health worker performance have evolved over time, shifting from one-time educational interventions, such as trainings and workshops, to multifaceted and ongoing approaches that incorporate post-training supervision, mentorship, and resource allocation [12, 13]. In line with this evolution in thinking, researchers have suggested the concept of individual-level capacity building should include technical capacity (knowledge and skills) but also intrapersonal capacity (motivation and confidence) and interpersonal capacity (leadership and management) [14].

Several forms of capacity-building interventions have been developed since Ethiopia’s HEP was established [5, 15–20]. These interventions have mainly been run by the government and partner organizations as part of HEP delivery, with the purpose of strengthening HEWs’ ability to deliver initial service packages or providing trainings on newly added services. Independent researchers have studied capacity-building interventions for HEWs that include training, supervision, supportive supervision, mentoring, and job aids [21–24]. However, there is limited evidence that maps the types of capacity-building interventions being used or the competencies targeted. Therefore, the aim of this scoping review was to systematically identify and map available evidence on the types of capacity-building interventions developed for HEWs in Ethiopia and the competencies they target, as well as to explore how successful the interventions have been. Findings from this review could inform government, partner organization, and other stakeholders about future focus areas for HEW capacity building.

### Review questions

1. What type of capacity-building interventions have been delivered to HEWs in Ethiopia?
2. What type of competencies have been addressed in these capacity-building interventions?
3. How successful the interventions have been?

## Methods

Because of the wide range of literature on capacity-building interventions and the diversity of outcomes investigated, a scoping review methodology was applied to answer the review questions. This methodology is applicable to any type of study design and publication type [25]. However, in this review we considered only studies with experimental, quasi-experimental, and cohort (prospective /retrospective) study designs, as one of the main review questions is related to the effects of capacity-building interventions. The review was conducted following the JBI methodology for scoping reviews [26]. The review protocol was not registered anywhere, as our review progressed to the data extraction phase before we considered protocol registration. The review framework, in terms of inclusion and exclusion criteria, is described in Table 1.

**Table 1:** Framework for review of capacity-building interventions designed for Ethiopian health extension workers (HEWs): Inclusion and exclusion criteria.

Review Framework/Content	Inclusion Criteria	Exclusion Criteria
Population	HEWs in Ethiopia	Other community health workers or assistants to HEWs (such as women development armies)
Intervention	Capacity-building interventions aimed at strengthening Ethiopian HEWs’ competencies at the individual level	Capacity-building interventions aimed at the community level
Comparator	No intervention	Not applicable
Outcome	Immediate outcomes, such as change in HEW knowledge, skills, or motivation; and/or intermediate outcomes, such as health service utilization among community members	Outcomes measured at the community level and not linked to the intervention provided to HEWs
Context	Ethiopia	Other countries
Study design	Experimental designs, quasi-experimental designs with at least two time point measurements before and after the intervention, and prospective or retrospective cohort designs conducted in Ethiopia	Cross-sectional designs, case-control studies, reviews
Publication type	Peer-reviewed published research articles, unpublished studies, the grey literature (such as organizational reports and government documents, if they report on interventions along with comparators or base- and endline assessments)	Abstracts and conference proceedings
Publication year	Published since 2003 (when Ethiopia’s Health Extension Program was established)	Publications before 2003
Languages	English	Languages other than English

### Search strategy and databases

First, an initial search was conducted on PubMed to identify published articles on the topic. The words used in the titles and abstracts of the relevant articles were used to develop a full search strategy for PubMed. The search strategy was developed by combining keywords (health extension worker, capacity building, competencies). Similar possible terminologies for these three keywords were considered in developing the search strategy. The search strategy was then customized for Embase and Scopus (see S1 File). Google Scholar was searched for unpublished studies and grey literature, as we wanted to investigate interventions to enhance HEW competencies regardless of publication status. The first 10 pages of Google Scholar results were screened for relevant literature on the topic. The reference lists for all relevant articles were also screened to identify additional articles eligible for inclusion.

### Study selection

First, all articles found during the literature search were imported to EndNote version 9. Duplicate articles were identified and removed electronically using EndNote’s “find duplicates” tool. Next, articles not in English, study protocols, review articles, and overviews were removed. Titles and abstracts were screened by two authors (TA and HM) based on the predefined inclusion and exclusion criteria described in Table 1.

### Data extraction

Data were independently extracted from individual articles by two reviewers (TA and HM) using a data extraction template. Data items were structured to capture the key elements of the review (intervention type, primary outcome in terms of capacity domain, main findings) along with other study characteristics (study design, study setting). Main findings, in terms of intervention effectiveness, were extracted as described in the Results section of the reviewed articles (e.g., percentage change after intervention, accompanying confidence intervals, p-values when provided). Data were initially extracted into a Microsoft Excel spreadsheet. The data extracted by the two independent reviewers were cross-checked by TA, and differences were resolved through discussion with the second data extractor (HM).

### Data synthesis

We used a categorization approach to synthesize the data. Main findings were categorized by capacity-building intervention, in response to the first review question. The second review question was also addressed using the aforementioned categories. Effects of capacity-building interventions were described in terms of percentage change in outcomes, as presented in the studies reviewed.

## Results

### Study selection

**Fig 1:**
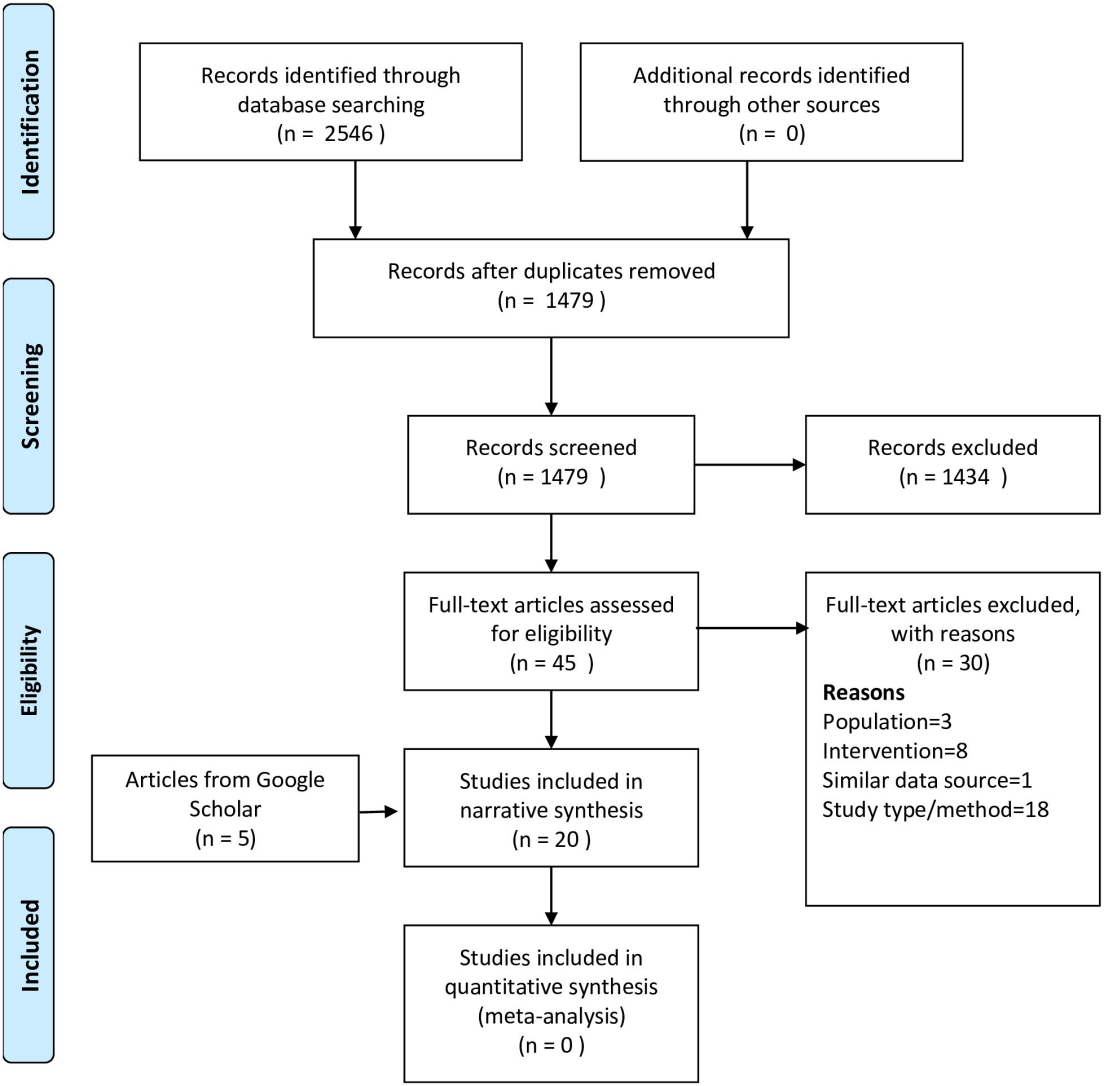
PRISMA flow diagram for database searches and study selection.

In total, 2,546 articles were identified from the three electronic databases (PubMed, Embase and Scopus), 1,479 were eligible for title and abstract screening, and 45 were eligible for full-text review (Fig 1). Of these 45 articles, 15 met our inclusion criteria. Additionally, by using Google Scholar and screening the reference lists of relevant articles, five additional articles that met our inclusion criteria were found. In total, 20 articles were eligible for full data extraction and analysis.

### Characteristics of included articles

Of the 20 articles, 12 measured outcomes in terms of percentage change pre- and post- intervention [17–19, 21–24, 27–31], 3 presented the results of randomized controlled trials [20, 28, 32], and the remaining 5 had a retrospective cohort design [33–37]. Of the 5 studies with a retrospective cohort design, 4 involved analyses of data gathered from longitudinal program monitoring [33–35, 37]. All studies considered rural HEWs.

Of all the capacity-building interventions rolled out to enhance HEWs’ competencies, training was the most studied. Of the 20 articles reviewed, 15 (75%) concerned training [17–19, 21–24, 27–31, 34, 36, 38]. Of these 15 articles, 3 featured interventions including equipment and supervision in addition to training [18, 22, 34], and 1 featured an intervention that trained both HEWs and other staff [21]. Three of the 15 articles described multi-level interventions, including interventions aimed at building capacity at the primary healthcare level and engaging the community [17, 19, 27]. Supportive supervision interventions were studied in 2 of the 20 articles [33, 37]. Finally, 1 of the 20 articles studied performance reviews and clinical mentoring meetings, alongside follow-up training and supervision [35]. Twelve studies measured knowledge and skill as an outcome. Only one study assessed whether capacity-building interventions improved inter- and intra-personal domains of capacity/competency. The effect of each capacity-building intervention is summarized in Table 2 and discussed in greater detail below.

**Table 2:** Summary of findings from studies of capacity-building interventions designed for Ethiopian health extension workers (HEWs).

First Author	Publication Year	Study Location/Region	Study Setting/Facility	Study Design	Intervention Type/Capacity-Building Domain	Competency Measured	Primary Outcome(s)	Main Findings
Ameha A	2014	Amhara, Oromia, SNNPR, Tigray	Health posts	Retrospective cohort	Supportive supervision	Skill	Consistency of iCCM skills of HEWs	Health post consistency for pneumonia, malaria, and diarrhea management increased by 3.0, 2.7 and 4.4-fold respectively, with non- overlapping CIs.
Ameha A	2019	Amhara, Oromia, SNNPR, Tigray	Health posts	Retrospective cohort	Training + equipment + supervision	Service utilization	Service utilization for young infants/neonates and children	21-fold increase in number of sick young infants seen at health post Mean number of sick neonates seen per year per health post: pre-CBNC intervention, 1.2 (95% CI: 0.63, 1,84); post-CBNC intervention, 26.4 (95% CI: 25.4, 27.3)
Atnafu A	2017	SNNPR	Community	Randomized controlled trial	Equipment (mobile phone)	Service utilization	Maternal and child health indicators	HEWs’ antenatal care follow-up at mothers’ homes increased from 5.21% at baseline to 29.75% after 13 months of intervention Significant increase in proportion of mothers receiving >4 antenatal care visits in the two intervention areas (Z = 17.6, *P* = 0.001 and Z = 4.04, *P* = 0.001) Reduced home deliveries in intervention areas (from 50.70% to 35.82% and from 61.57% to 33.73% for the two areas)
Berhanu D	2021	Tigray, Amhara, SNNPR, Oromia	Community	Pre- and post-intervention study	Training + equipment + supervision	Service utilization	19 indicators from 9 different program components	Proportion of women who reported ≥1 antenatal care visit increased by 15 percentage points (95% CI: 10, 19; *P* <0.0001) Proportion of women with ≥4 antenatal care visits increased by 17 percentage points (95% CI: 13, 22; *P* <0.0001) Institutional delivery rate increased by 40 percentage points (95% CI: 35, 44) Proportion of mothers with young infants with possible serious bacterial infections who reported their child received amoxicillin for 7 days increased by 50 percentage points (95% CI: 37, 62) and proportion who reported receiving gentamicin for 7 days increased by 15 percentage points (95% CI: 5, 25) Concurrent use of both antibiotics increased by 12 percentage points (95% CI: 4, 19)
Berhanu D	2020	Oromia, Amhara, Tigray, SNNPR	Health post and community	Pre- and post-intervention study	1) community engagement, 2) primary care–level capacity building, 3) ownership and accountability for child health services at district level	Service utilization	Child health service utilization	No evidence to suggest intervention increased care utilization for sick children. Care-seeking for any illness in the 2 weeks before the survey for children aged 2–59 months at baseline was 58% (95% CI 47 to 68) in intervention and 49% (95% CI 39 to 60) in comparison areas. At end-line it was 39% (95% CI 32 to 45) in intervention and 34% (95% CI 27 to 41) in comparison areas (difference-in-differences -4 percentage points, adjusted OR 0.49, 95% CI 0.12 to 1.95).
Daka D	2023	Oromia, Amhara, Tigray, SNNPR	Health post and community	Pre- and post-intervention study	1) community engagement, 2) primary care–level capacity building, 3) ownership and accountability of child health services at district level	Skill	% of sick children managed according to guidelines	Intervention not associated with improved quality of HEWs’ management of sick children for cough (DiD = -21%, *P* = 0.55) or malnutrition (DiD = 5%, *P* = 0.16)
Datiko D	2009	SNNPR	Community	Randomized controlled trial	Training	Skill	Case notification rate, treatment success rate	Higher mean case detection rate in control areas (122.2% vs 69.4%, *P* <0.001) Higher mean treatment success rate in intervention areas (89.3% vs 83.1%, *P* = 0.012)
Datiko D	2017	SNNPR	Community	Pre- and post-intervention study	Training	Skill	TB case notification rate, treatment success rate	Smear-positive TB case notification rate increased from 64 (95% CI: 62.5, 65.8) to 127 (95% CI: 123.8, 131.2) per 100,000 population. In subsequent years, smear-positive case notification rate declined by 9%/year. No change in case notification rate in control area. All forms of TB case notification rate per 100,000 population increased from 102 (95% CI: 99.1, 105.8) to 177 (95% CI: 172.6, 181.0) in the first year of intervention. Treatment success rate for all forms of TB was 76% pre-intervention (2358/3110 cases) and 95% post-intervention (19,003/20,108 cases; *P* <0.001) Proportion of patients lost to follow-up decreased from 21% pre-intervention (651/3110 cases; 95% CI 19.5%, 22.4%) to 3% post-intervention (598/20,108 cases; 95% CI: 2.7%, 3.2%; *P* <0.001)
Deborah S	2020	Oromia	Health post and community	Quasi-experimental study	Training, materials (modified maternal-and-child-health card and tally sheet), supportive supervision on post-partum family planning	Service utilization	Service utilization	Women who delivered at home in intervention arm were 45% more likely to adopt contraception (adj. HR = 1.45, 95% CI: 1.01, 2.07)
Getachew T	2021	SNNPR	Community	Randomized controlled trial	Training, supportive supervision, HEW performance reviews	Skills	Skills	Intervention not associated with improved classification of childhood illnesses by HEWs. Difference-in-differences was 6% for correct classification of fever or malaria [aOR = 1.45 95% CI: 0.81–2.60], 4% for respiratory tract infection [aOR = 1.49 95% CI: 0.81–2.74], and 5% for diarrheal diseases [aOR = 1.74 95% CI: 0.77–3.92].
Gezmu T	2021	SNNPR	Health posts	Pre- and post-intervention study with comparison group	Training	Service provision	Scabies load	Scabies cases declined in intervention district from 7.6 to 1.6 per 1,000 population (4.8-fold reduction). In control district, scabies cases increased from 1.3 to 2.4 per 1,000 population (1.8-fold increase). In intervention district, proportion of scabies patients with secondary skin infections fell from 78% (1,227/1,565 cases) to 48% (156/326 cases), *P* <0.001. In control district, difference was insignificant: 14% (39/288) to 17% (86/521), *P* = 0.2.
Lynn M	2014	Amhara and Oromia	Health post and community	Pre- and post-intervention study	Multiple interventions (training for HEWs, traditional birth attendants, women development army); behavior change communication for community members	Skills and confidence	skills, confidence	Demonstrated increased ability and self-reported confidence to provide this care. Mean postintervention scores for health extension workers, community health development agents, and traditional birth attendants were 72% to 80% and greater than pre-training scores (all P < .001)
Mengesha W	2018	SNNPR	Health post	Pre- and post-intervention study	Training + equipment	Skill	Quality of data	Increased completeness & quality of data (percentage and CIs not provided)
Miller N	2016	Oromia	Health post	Retrospective cohort	Quality improvement interventions (PRCMM, supportive supervision, follow-up training)	Skill	Proportion of children correctly managed for major iCCM illnesses	Children managed by a HEW who attended PRCMM had 8.3 (95% CI: 2.34, 29.51) times the odds of being correctly managed than children managed by an HEW who did not attend Management by a HEW who received follow-up training significantly increased odds of correct management (OR = 2.09, 95% CI: 1.05, 4.18) Supervision of iCCM did not significantly affect odds of receiving correct care (OR = 0.63, 95% CI: 0.23, 1.72)
Swanson V	2021	Amhara	Health post	Pre- and post-intervention study	Behavior-change skill enhancement training	Skill and knowledge	Behavioral change, health outcomes attained, learning achieved	Positive impact on HEWs’ skills, knowledge, and motivation to change women’s nutritional behavior (all *P* <0.001)
Tesfau Y	2022	Tigray	Community	Pre- and post-intervention study	Training (includes training other types of staff)	Service utilization	Percentage of women and/or newborns visited at home ≤3 days after delivery	Average 23.5% increase in postnatal health visit coverage within 3 days (DiD, *P* <0.001) Knowledge of at least three danger signs increased by 13.6% (*P* = 0.012) Average 27.6% increase in checking mothers for heavy bleeding (DiD, *P* = 0.011) Maternal blood pressure checks increased from 9.4% to 93.3% in intervention district, 5.8% to 11.8% in comparison districts, indicating a statistically significant contribution of 77.9% by the intervention (*P* <0.001) Increase in average knowledge of cord bleeding/presence of pus 15% (DiD, *P* = 0.004), low body weight/pre-term 16% (DiD, *P* <0.001), low body temperature 11.4% (DiD, *P* = 0.003), and fever 13.3% (DiD, *P* = 0.023) 9% difference in clean cord-care practices (*P* = 0.025). Statistically significant increase in skin-to-skin care (12.2%, *P* = 0.022), borderline significant increase in early initiation of breastfeeding (10.5%, *P* = 0.051)
Tessema M	2013	Amhara	Health post	Pre- and post-intervention study	Training	Knowledge	HEWs’ knowledge of community-based nutrition	Significant knowledge change observed after training (p<0.05)
Tilahun D	2019	SNNPR	Health post	Retrospective cohort	HEAT	Belief	HEWs’ positive and negative beliefs, social distancing regarding autism	Both HEAT (*P* <0.001) and HEAT+ group (*P* <0.001) showed decreased social distance toward children with autism HEAT-trained (Z = -6.14, r = -0.42, *P* <0.001) and HEAT+ trained (Z = -6.24, r = -0.44, *P* <0.001) HEWs were more likely to believe that children with autism can improve their language skills
Tiruneh G	2019	Amhara, Oromia, SNNPR, Tigray	Health post	Retrospective cohort	Supportive supervision	Skill	Consistency of HEW neonatal sepsis management skills	Consistency of HEW sepsis management skills was not different between health posts visited once vs twice; however, difference was statistically significant between those visited once vs more than twice (OR 2.49; *P* <0.05)
Yassin M	2013	SNNPR	Community	Pre- and post-intervention study	Training	Skill	TB case notification rate, treatment outcomes	Case notification rate in intervention zone increased from 64 PTB+ cases per 100,000 population/year (95% CI: 62.5, 65.8) to 127 (95% CI: 123.8, 131.2) Notification rates for all forms of TB increased from 102 cases per 100,000 population (95% CI: 99.1, 105.8) to 177 (95% CI: 172.6, 181.0); p-value not provided Treatment success rate increased from 77% to 93% (*P* <0.05), defaulter rate decreased from 11% to 3% (*P* <0.05)

**Abbreviations**: CBNC, community-based newborn care; CI, confidence interval; CMNH, child, maternal, and newborn health; CoS, consistency of skills; DiD, difference-in-difference; HEAT, health education and training; HEAT+, health education and training and video-based training; HR, hazard ratio; iCCM, integrated community case management of childhood illness; OR, odds ratio; PRCMM, performance review and clinical mentoring meeting; PTB+, smear-positive pulmonary tuberculosis; SNNPR, Southern Nations, Nationalities and Peoples Region; TB, tuberculosis.

### Training

Of the 15 articles that considered training as a capacity-building intervention, 7 assessed the exclusive effect of HEW training on immediate or intermediate outcomes [24, 28–31, 36, 38]. One article measured morbidity reduction following HEW training on scabies case management [29], which could be considered an ultimate outcome. The training interventions studied were face-to-face and ranged from 2 to 14 days. One article described a non-disease-specific HEW training intervention to improve communication skills [30]. The other interventions trained HEWs on managing, detecting, screening, and creating awareness for specific diseases. After training interventions were implemented, articles reported increased case notification and treatment success rates [24, 28, 38], numbers of sick young infants seen at health posts [34], antenatal and delivery care service utilization [18], postpartum family planning utilization [22], completeness and quality of data [23], and HEW knowledge, skills, and motivation to change women’s behavior [30]. They also reported significant increases in HEW knowledge and practice regarding danger signs during delivery and newborn care practices [21], developing a positive attitude [36], and reducing morbidity [29].

Two articles assessed interventions that included HEW training, as well as supplying essential commodities and supervision, to improve the national community-based newborn care program [18, 34]. Because of this, in these studies HEW training should be considered one contributing factor to the increase observed in service utilization and healthcare-seeking behavior following implementation of the intervention. Likewise, the significant increase in postnatal care utilization observed in another study reflects multiple training interventions targeted to nurses, midwives, and district health officers, as well as HEWs [21]. Two articles studied the effect of multiple interventions (community engagement, primary care–level capacity building, and ownership and accountability of child health services at the district level), in addition to HEW training [17, 27]. This approach was not associated with improved quality of HEWs’ management of sick children, and no evidence was reported to suggest the interventions increased care utilization for sick children. In addition, one study revealed that an intervention that included HEW training, supportive supervision, and performance reviews did not improve HEWs’ classification of childhood illnesses [20].

### Supportive supervision

The effect of supportive supervision interventions on HEW competencies was studied in two articles [33, 37]. In these articles, supportive supervision was conceptualized as a visit by a supervisor to health posts to coach HEWs and ensure proper case management, promote use of job aids, provide problem-solving to encourage and motivate HEWs to improve their performance, and support data collection to monitor and evaluate programs. Both studies used longitudinal program monitoring data to demonstrate that supportive supervision improved the consistency of integrated community case management of childhood illness (iCCM) skills [33] and neonatal sepsis management skills [37]. Another article demonstrated that supportive supervision did not significantly affect the odds of HEWs correctly managing common childhood illnesses [35].

### Performance review and clinical mentoring meetings

Only one article assessed the effect of performance review and clinical mentoring meetings (PRCMMs) on iCCM [35]. This study considered PRCMMs as part of a quality improvement intervention that also included follow-up training and supervision, but measured the independent effect of PRCMM on iCCM. It showed that children managed by HEWs who attended PRCMMs had higher odds of being correctly managed. Management by HEWs who received follow-up training also significantly increased the odds of correct disease management.

### Equipment supply

One article assessed the effect of distributing mobile phones equipped with the FrontlineSMS app to HEWs [32]. Using this app, maternal-, child-, and stock-related forms are submitted to a central server, which in turn sends reminders about scheduled antenatal care visits, expected delivery dates, postnatal care, immunization schedules, and vaccine and contraceptive stock status. This intervention improved the proportion of mothers receiving more than four antenatal care visits, rates of antenatal care visits delivered by HEWs, and proportion of deliveries attended by skilled health workers, while significantly reducing stockouts of preferred contraceptive products. However, there was no statistical difference in family planning utilization and child immunization.

## Discussion

In this scoping review, we found capacity-building interventions designed for HEWs were largely disease-specific. The most common capacity-building intervention studied was training, followed by supportive supervision, performance review and clinical mentoring meetings, and equipment supply; the most salient domains of capacity investigated at the HEW level were knowledge and skill. Articles reported that capacity-building interventions for HEWs significantly improved both immediate outcome (knowledge, skills, attitude change among HEWs) and intermediate outcomes (increased service utilization and health-seeking behavior among community members). However, not all studies reported positive results. Based on the results of our review, training is likely a beneficial capacity-building intervention to improve HEWs’ knowledge, skills, and attitudes. This finding is consistent with evidence from other countries that demonstrates the importance of training in improving CHWs’ knowledge, attitude, practice/skills, and confidence [39–47].

The 2019 National Assessment of Ethiopia’s HEP [48] indicated that a mismatch remains between the current capacity of HEWs and the skills required to meet the needs of the community and effectively deliver expanded HEP service packages. To address this mismatch, in-service training will likely continue to be used as a capacity-building intervention. In line with other planned workforce development interventions, Ethiopia’s national roadmap for optimizing the HEP prioritizes building HEW capacity through upgraded trainings and on-the-job coaching [49]. However, in addition to training, a broader and more comprehensive set of individual capacity-building initiatives will be necessary to address the mismatch between the current capacity of HEWs and the skills they require. HEWs typically work in challenging environments [48, 50]. A high proportion express the intention to leave their positions [51, 52], they report low levels of motivation and satisfaction with their career [53–56], and they are at high risk for burnout [57]. Therefore, addressing these issues in capacity-building initiatives will be important for improving HEWs’ long-term performance. In addition, as a recent systematic review suggests [14], interventions to strengthen individual-level capacity should be conceptualized in terms of targeting a combination of technical ability (knowledge and skill), intrapersonal capacity (motivation and confidence), and interpersonal capacity (leadership and management).

Supportive supervision was the other key capacity-building strategy studied in the articles reviewed. Although only two studies assessed the effect of supportive supervision on HEWs’ disease management skills [33, 37], the results were promising and also consistent with previous findings in this area. One review has shown that among primary healthcare workers in low- and middle-income countries, supportive supervision is linked to improved quality of care for services such as immunization, malaria management, and childhood diarrhea management [13]. A longitudinal study performed in India showed that supportive supervision improves CHW performance to deliver services as per the nutrition program guideline [58], and a randomized controlled trial performed in Pakistan showed that it improves CHW community case management skills [59]. Unlike traditional supervision, which is prone to fault finding, supportive supervision focuses on identifying and resolving problems, as well as providing constructive feedback; it involves an ongoing positive relationship between supervisee and supervisor [13, 60, 61]. This kind of supervision is effective beyond building technical capacity, having been shown to improve CHW motivation and provide CHWs with guidance for solving problems and assistance with managing resources and logistics [13, 62]. Both the articles we reviewed in this study used longitudinal program monitoring data to assess the effect of supportive supervision on HEWs’ disease management skills. Although such program data are informative, a robust study design that compares and contrasts different supportive supervision strategies and their respective influences on HEW performance and motivation could be helpful.

In the real world, and particularly in Ethiopia’s HEP, performance reviews, clinical mentoring meetings, and supportive supervision are implemented concurrently, alongside other overlapping capacity-building activities. One study we reviewed measured the effect of these quality improvement interventions within Ethiopia’s national iCCM program [35]. The results suggested that PRCMM and follow-up training were effective interventions for HEWs, whereas supportive supervision did not affect rates of correct management of childhood illnesses. Given this finding, which contradicts other findings on the beneficial effects of supportive supervision on disease management within the same program [33, 37], further studies, conducted independently of these national programs, will be important for gathering stronger evidence about the effectiveness of supportive supervision.

### Strengths

Our search strategy considered numerous terminologies in order to capture different capacity-building activities, such as trainings, mentorship, supervision, and coaching, as well as multiple terminologies for competency domains that extended from knowledge and skills to leadership and management.

### Conclusions

In this scoping review, we mapped available evidence on the type of capacity-building interventions being implemented for Ethiopian HEWs and the competencies targeted by these interventions. Our review reveals that various forms of capacity-building interventions have been developed to improve HEW competencies, such as trainings, supportive supervision, and equipment supply. However, these interventions mainly focus on improving technical competencies, such as knowledge and skills related to specific diseases or health problems. Such approaches, though crucial, may not improve HEW performance in a sustainable fashion. For this reason, a comprehensive set of capacity-building initiatives that addresses HEWs’ motivation, job satisfaction, communication skills, commitment, and resource allocation is necessary.

## Supporting information

S1 File Search strategies applied for the three electronic databases used in this study. (DOCX)

S2 File Completed PRISMA 2020 checklist. (DOCX)

S3 File Self-developed data extraction form. (DOCX)
