# Supplementary material for: Capacity-building interventions for health extension workers in Ethiopia: A scoping review
Source: PLoS One. 2025 Jan 13;20(1):e0317198. doi: 10.1371/journal.pone.0317198 (PMC11729990; doi:10.1371/journal.pone.0317198)
Supplement: S1 File — (DOCX) [file pone.0317198.s001.docx]

**Search terms: PubMed**

**Population:** “health extension worker*” or HEW or HEWs or “health extension program*” or HEP or HEPs or “community health worker*” or CHW or CHWs or “community-based health worker*” or “community-based health program*”

**Intervention:** train* or supervision or “supportive supervision” or education or mentor* or empower* or intervention* or strategy* or approach* or advocacy or tool* or message* or guideline* or “treatment guideline*” or equipment* or support* or program* or “capacity building” or “capacity building program*” or “capacity strengthening” or “technical assistance*” or “professional development*” or “capacity develop*” or coach*

**Comparator:** No intervention

**Outcome:** knowledge or skill* or motivation or attitude* or behavior* or perception* or belief* or “client satisfaction*” or “patient satisfaction*” or satisfaction or screen* or communication or counseling or coverage* or “service utilization” or “service delivery” or “service provision*” or performance* or commitment or “case management” or “case finding” or “case detect*” or “case notification” or detect* or “treatment outcome*” or management or leadership or governance or “service quality” or “health care behavior*” or “help-seeking behavior*” or “health care practice*” or consultation* or treat* or classify* or classification or identify* or identification or “disease classification” or assess* or “guideline adherence” or adherence or accuracy or completeness or “delivery of care” or access or competency or competencies or capacity or capacities or “service improvement*” or ability or abilities or practice* or self-efficacy or confidence or conflict resolution or timeliness

**Context:** Ethiopia*

| Database | Search Strategies | Accessed articles | Date |
| --- | --- | --- | --- |
| **Scopus**  #1 | TITLE-ABS-KEY ( "health extension worker*"  OR  hew  OR  hews  OR  "health extension program*"  OR  hep  OR  heps  OR  "community health worker*"  OR  chw  OR  chws  OR  "community-based health worker*"  OR  "community-based health program*" ) | 69,843 | 17/05/2023 |
| #2 | TITLE-ABS-KEY ( train*  OR  supervision  OR  "supportive supervision"  OR  education  OR  mentor*  OR  empower*  OR  intervention*  OR  strategy*  OR  approach*  OR  advocacy  OR  tool*  OR  message*  OR  guideline*  OR  "treatment guideline*"  OR  equipment*  OR  support*  OR  program*  OR  "capacity building"  OR  "capacity building program*"  OR  "capacity strengthening"  OR  "technical assistance*"  OR  "professional development*"  OR  "capacity develop*" ) | 25,607,553 | 17/05/2023 |
| #3 | TITLE-ABS-KEY ( knowledge  OR  skill*  OR  motivation  OR  attitude*  OR  behavior*  OR  perception*  OR  belief*  OR  "client satisfaction*"  OR  "patient satisfaction*"  OR  satisfaction  OR  screen*  OR  communication  OR  counseling  OR  coverage*  OR  "service utilization"  OR  "service delivery"  OR  "service provision*"  OR  performance*  OR  commitment  OR  "case management"  OR  "case finding"  OR  "case detect*"  OR  "case notification"  OR  detect*  OR  "treatment outcome*"  OR  management  OR  leadership  OR  governance  OR  "service quality"  OR  "health care behavior*"  OR  "help-seeking behavior*"  OR  "health care practice*"  OR  consultation*  OR  treat*  OR  classify*  OR  classification  OR  identify*  OR  identification  OR  "disease classification"  OR  assess*  OR  "guideline adherence"  OR  adherence  OR  accuracy  OR  completeness  OR  "delivery of care"  OR  access  OR  competency  OR  competencies  OR  capacity  OR  capacities  OR  "service improvement*" ) | 43,156,330 | 17/05/2023 |
| #4 | ALL ( ethiopia ) | 314,076 | 17/05/2023 |
| #5 | #1 AND #2 AND #3 AND #4 | 1,813 | 17/05/2023 |
| EMBASE  #1 | 'health extension worker'/exp OR 'health extension worker' OR 'health extension program':ab,ti OR 'community health worker':ab,ti OR 'community-based health worker':ab,ti OR 'community-based health program':ab,ti | 2,063 | 17/05/2023 |
| #2 | 'training'/exp OR training OR supervision:ab,ti OR 'supportive supervision':ab,ti OR education:ab,ti OR mentor:ab,ti OR 'empower':ab,ti OR intervention:ab,ti OR strategy:ab,ti OR approach:ab,ti OR advocacy:ab,ti OR tool:ab,ti OR message:ab,ti OR guideline:ab,ti OR 'treatment guideline':ab,ti OR equipment:ab,ti OR support:ab,ti OR program:ab,ti OR 'capacity building':ab,ti OR 'capacity building program':ab,ti OR 'capacity strengthening':ab,ti OR 'technical assistance':ab,ti OR 'professional development':ab,ti OR 'capacity development':ab,ti | 7,462,875 | 17/05/2023 |
| #3 | 'training'/exp OR training OR supervision:ab,ti OR 'supportive supervision':ab,ti OR education:ab,ti OR mentor:ab,ti OR 'empower':ab,ti OR intervention:ab,ti OR knowledge:ab,ti OR skill:ab,ti OR attitude:ab,ti OR behavior:ab,ti OR perception:ab,ti OR belief:ab,ti OR 'client satisfaction':ab,ti OR 'patient satisfaction':ab,ti OR satisfaction:ab,ti OR screen:ab,ti OR 'communication':ab,ti OR counseling:ab,ti OR coverage:ab,ti OR 'service utilization':ab,ti OR 'service delivery':ab,ti OR 'service provision':ab,ti OR performance:ab,ti OR commitment:ab,ti OR 'case management':ab,ti OR 'case finding':ab,ti OR 'case detect':ab,ti OR 'case notification':ab,ti OR detect:ab,ti OR 'treatment outcome':ab,ti OR management:ab,ti OR leadership:ab,ti OR governance:ab,ti OR 'service quality':ab,ti OR 'health care behavior':ab,ti OR 'help seeking behavior':ab,ti OR 'health care practice':ab,ti OR consultation:ab,ti OR treat:ab,ti OR classify:ab,ti OR classification:ab,ti OR identify:ab,ti OR identification:ab,ti OR 'disease classification':ab,ti OR 'assess':ab,ti OR 'guideline adherence':ab,ti OR adherence:ab,ti OR accuracy:ab,ti OR completeness:ab,ti OR 'delivery of care':ab,ti OR access:ab,ti OR competency:ab,ti OR competencies:ab,ti OR capacity:ab,ti OR capacities:ab,ti OR 'service improvement':ab,ti | 12,976,159 | 17/05/2023 |
| #4 | 'ethiopia'/exp OR ethiopia OR ethiopian | 42,453 | 17/05/2023 |
| #5 | #1 AND #2 AND #3 AND #4 | 161 | 17/05/2023 |

SCOPUS final link: [Scopus - Document search results (uq.edu.au)](https://www-scopus-com.ezproxy.library.uq.edu.au/results/results.uri?origin=searchhistory&sort=plf-f&src=s&mltEid=&mltAll=t&sid=b817aa13158aaf89a9626017ef819a4f&sot=comb&sdt=comb&sl=1568&s=%28TITLE-ABS-KEY%28%22health+extension+worker*%22+or+HEW+or+HEWs+or+%22health+extension+program*%22+or+HEP+or+HEPs+or+%22community+health+worker*%22+or+CHW+or+CHWs+or+%22community-based+health+worker*%22+or+%22community-based+health+program*%22%29%29+AND+%28TITLE-ABS-KEY%28train*+or+supervision+or+%22supportive+supervision%22+or+education+or+mentor*+or+empower*+or+intervention*+or+strategy*+or+approach*+or+advocacy+or+tool*+or+message*+or+guideline*+or+%22treatment+guideline*%22+or+equipment*+or+support*+or+program*+or+%22capacity+building%22+or+%22capacity+building+program*%22+or+%22capacity+strengthening%22+or+%22technical+assistance*%22+or+%22professional+development*%22+or+%22capacity+develop*%22%29%29+AND+%28TITLE-ABS-KEY%28knowledge+or+skill*+or+motivation+or+attitude*+or+behavior*+or+perception*+or+belief*+or+%22client+satisfaction*%22+or+%22patient+satisfaction*%22+or+satisfaction++or+screen*+or+communication+or+counseling+or+coverage*+or+%22service+utilization%22+or+%22service+delivery%22+or+%22service+provision*%22+or+performance*+or+commitment+or+%22case+management%22+or+%22case+finding%22+or+%22case+detect*%22+or+%22case+notification%22+or+detect*+or+%22treatment+outcome*%22+or+management+or+leadership+or+governance+or+%22service+quality%22+or+%22health+care+behavior*%22+or+%22help-seeking+behavior*%22+or+%22health+care+practice*%22+or+consultation*+or+treat*+or+classify*+or+classification+or+identify*+or+identification+or+%22disease+classification%22+or+assess*+or+%22guideline+adherence%22+or+adherence+or+accuracy+or+completeness+or+%22delivery+of+care%22+or+access+or+competency+or+competencies+or+capacity+or+capacities+or+%22service+improvement*%22%29%29+AND+%28ALL%28Ethiopia%29%29&txGid=d162fd77c578b07fc18c375513748920)
EMBASE final link: <https://www.embase.com/#advancedSearch/resultspage/history.5/page.1/25.items/orderby.date/source>.
