# Supplementary material for: Capacity-building interventions for health extension workers in Ethiopia: A scoping review
Source: PLoS One. 2025 Jan 13;20(1):e0317198. doi: 10.1371/journal.pone.0317198 (PMC11729990; doi:10.1371/journal.pone.0317198)
Supplement: S3 File — (DOCX) [file pone.0317198.s003.docx]

Data extraction tool template

| **Title** | **First author** | **Publication year** | **Study design** | **Study setting/type of facility** | **Study location/region** | **Type of intervention** | **Type of competency measured** | **Primary outcome** | **Main findings** |
| --- | --- | --- | --- | --- | --- | --- | --- | --- | --- |
|  |  |  |  |  |  |  |  |  |  |
|  |  |  |  |  |  |  |  |  |  |
|  |  |  |  |  |  |  |  |  |  |
|  |  |  |  |  |  |  |  |  |  |
|  |  |  |  |  |  |  |  |  |  |
|  |  |  |  |  |  |  |  |  |  |
|  |  |  |  |  |  |  |  |  |  |
